# Mechanisms and potential therapeutic molecular targets in blood–brain barrier disruption following subarachnoid hemorrhage: a review of early brain injury

**DOI:** 10.3389/fneur.2025.1678839

**Published:** 2025-10-21

**Authors:** Hao Ouyang, Hua Gu, Yong Cai, Chenli Wang

**Affiliations:** ^1^The First People’s Hospital of Huzhou (The First Affiliated Hospital of Huzhou University), Huzhou, China; ^2^Zhejiang Chinese Medical University, Hangzhou, Zhejiang, China; ^3^Changsha Medical College, Changsha, Hunan, China

**Keywords:** hemorrhagic stroke, intracranial aneurysm, brain edema, neuron apoptosis, brain herniation, endothelial cells, pericytes, astrocytes

## Abstract

Subarachnoid hemorrhage (SAH) is a devastating stroke characterized by acute onset, severe symptoms, and a poor prognosis. A series of pathological changes occur within 72 h after SAH, leading to early brain injury (EBI). Blood–brain barrier (BBB) disruption is a key factor contributing to the EBI progression. When the BBB is compromised, detrimental substances and immune cells have the potential to infiltrate brain tissues, and a range of mechanisms contribute to the disruption of the BBB following SAH. This review provides a comprehensive overview of the current knowledge regarding the underlying mechanisms and potential therapeutic targets in BBB disruption during EBI following SAH. It focuses on the dysfunction of endothelial cells, tight junctions, astrocytes, and pericytes; the specific molecular targets for EBI after SAH; and new emerging treatments for BBB disruption in EBI after SAH.

## Introduction

Subarachnoid hemorrhage (SAH) is a critical form of hemorrhagic stroke resulting from the rupture of pathological blood vessels, leading to the direct influx of blood into the subarachnoid space within the brain (1). The prevalence of SAH was approximately 10 per 100,000 individuals in the general population, accounting for 5–10% of all stroke cases, while the incidences were more evident in specific countries (Finland and Japan), elderly, women, black races, family history of SAH and some heritable connective-tissue disorders (2, 3). An underlying intracranial aneurysm rupture is the primary etiology in approximately 80–85% of cases of spontaneous SAH (4). Brain injury induced by SAH could be divided into early brain injury (EBI) and delayed brain injury. EBI is the primary prognostic factor for aneurysmal SAH, which can occur within minutes to 72 h after the initial bleeding. EBI is characterized by increased intracranial pressure, resulting in global cerebral ischemia or the presence of extravasated blood (5, 6). Multiple physiological disturbances cause EBI, and various pathological changes occur within 72 h after SAH, potentially leading to secondary brain tissue damage (7). In clinical practice, surgical clipping or endovascular coiling used for early aneurysmal obliteration are widely applied to prevent rebleeding and improve the prognosis of SAH. Moreover, intensive medical care should be used to manage delayed cerebral ischemia after ruptured aneurysm is successfully treated, irrespectively for cerebral vasospasm (CVS), and other various medical complications status. Furthermore, it is crucial to closely monitor and effectively manage surrogate markers for early brain injury (EBI) in order to enhance the prognosis of subarachnoid hemorrhage (SAH). These markers include the initial clinical severity, extent of subarachnoid hemorrhage, and the presence of global cerebral edema (5).

Studies have shown that the disruption of the BBB is a significant factor in the development of brain edema caused by EBI (8). An increased intracranial pressure following SAH is strongly correlated with reduced cerebral blood flow and widespread cerebral ischemia. This is accompanied by the extravasation of blood and degradation products, which can impact the advancement of brain injury. The disruption of BBB permeability significantly contributes to death within 72 h after SAH, as it could induce brain edema, increase intracranial pressure, cause secondary neuron apoptosis, and result in brain herniation (9). The BBB in the cerebrovascular system is composed of neurons, endothelial cells, pericytes, astrocytes, microglia, and vascular smooth muscle cells (VSMC) (10). The BBB regulates brain homeostasis by restricting the entry of neurotoxic plasma components, blood cells, and pathogens from the systemic circulation (11). Preclinical SAH studies have demonstrated that BBB permeability increases at 24–36 h, peaks at 48 h, and normalizes at 72 h. Disruption of the BBB function is closely correlated with damage to the basal lamina and microvasculature (12, 13). Thus, inhibiting of BBB disruption could considered as an effective strategy to improve the prognosis of EBI after SAH.

Considering the complexity of SAH and the difficulty in identifying effective treatments, exploring new therapeutic approaches for EBI is crucial to improving the prognosis of SAH. Therefore, it is necessary to perform a thorough review of the literature in order to evaluate the underlying mechanisms and identify potential molecular targets for therapeutic interventions in BBB disruption following EBI after SAH.

## Mechanisms of EBI after SAH

**Figure 1:**
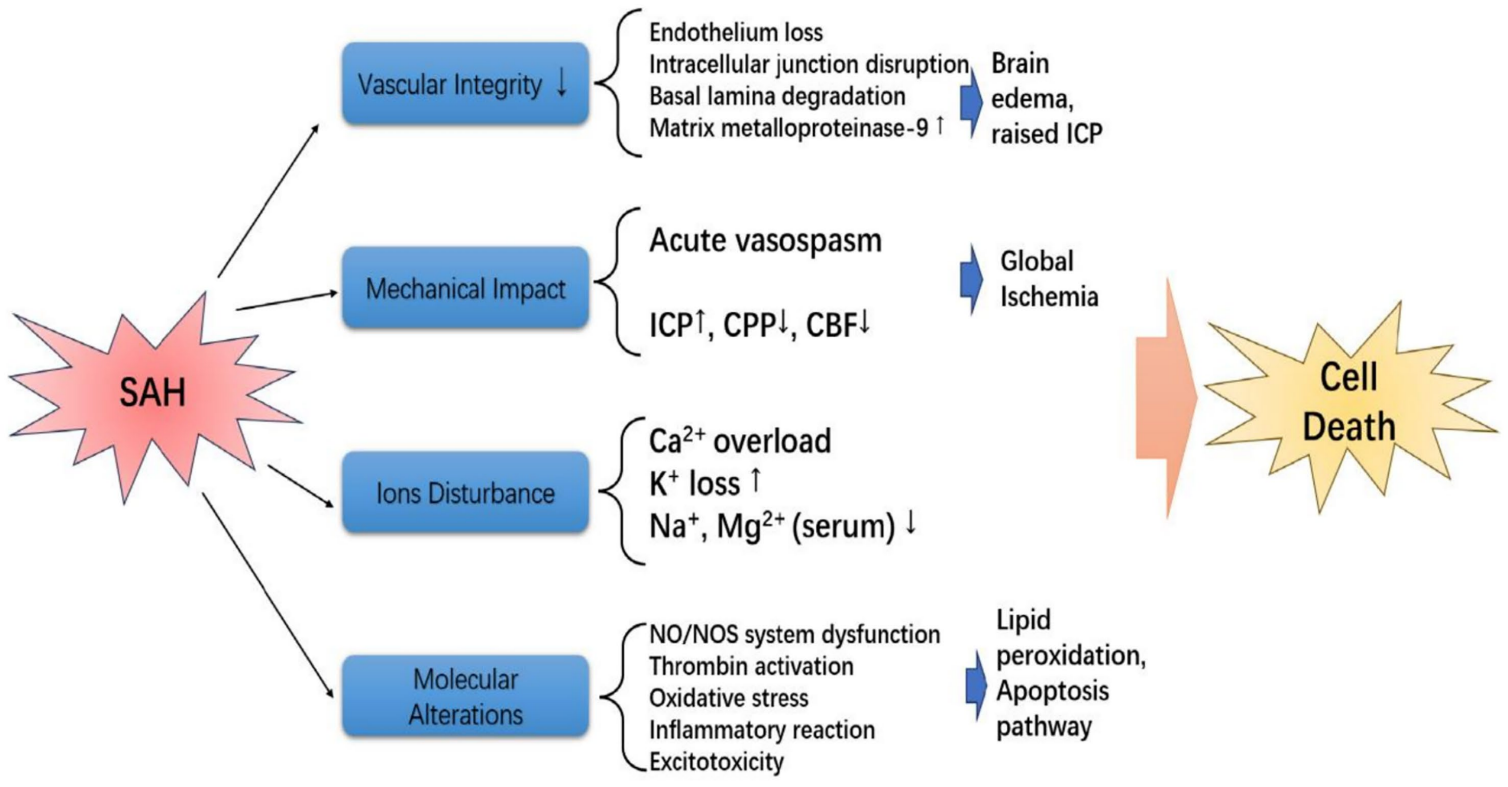
The mechanism of EBI after SAH, which mainly included vascular integrity, mechanical impact, ions disturbance, and molecular alterations, and these changes could induce cell death.

A intracranial pressure (ICP) peak after SAH could arrest in intracranial circulation (14), and severe global ischemic injury could caused by temporary intracranial circulatory arrest via hemostasis, which was associated with autoregulation loss, lower cerebral perfusion pressure (CPP) or cerebral blood flow (CBF), and elevated secondary ICP (15). Moreover, the cytotoxic edema could affect by hypoxic state via energy failure in neurons and glia (16). Furthermore, ischemia can trigger the apoptosis of BBB cells, leading to an augmented permeability of serum from the blood vessels into the cerebral tissues, which is primarily attributed to the death of endothelial cells and perivascular astrocytes (17). The astrocytes and microglia were activated after SAH, which could induce the up-regulation of pro-inflammatory cytokines, and then the brain parenchyma was affected (18). Finally, the pathophysiological changes during the EBI period after SAH within 72 h included raised ICP, reduced CPP and CBP, BBB disruption, acute vasospasm, autoregulation dysfunction, brain swelling, and brain edema (16). Multiple mechanisms have been shown to be involved in cell death and subsequent dysfunction following SAH, and the specific details are illustrated in Figure 1 (19–22). In summary, EBI after SAH is a synergistic outcome of multiple mechanisms, including elevated ICP, reduced CBF/CPP, ion disturbance, and molecular alterations. These processes collectively induce cell death in neurovascular units, laying the pathological foundation for BBB disruption. The details of mechanisms for EBI after SAH are summarized in Table 1.

**Table 1:** Potential mechanism for early brain injury after SAH.

Components	Molecular mediators	Targets
Vascular integrity	Endothelial cell apoptosis, TNF-α, IL-1β, and thromboxane A2	BBB disruption (23)
	MMP-9, TJ proteins between endothelial cells	BBB disruption (24, 28)
	AQP4	Cerebral edema (25)
Mechanical impact	CBF, ICP, and CPP	Brain’s microcirculation (12)
	MMP-9	Platelet aggregation (28)
	CMRO2	Oxygen and glucose metabolism (29)
Ions disturbance	Intracellular calcium, potassium, sodium, and magnesium	CSD (30–33)
Molecular alterations	Ras-MAPK-NF-κB, JAK/STAT, and TLR4/NF-κB	IL-1, IL-6, and TNF-α (35–37)
	Lipid peroxidation and protein oxidation	Oxidative stress (39)
	NOS	ROS, oxidative stress (40)

Among the multiple pathological processes of EBI, BBB disruption is a pivotal link connecting upstream insults (e.g., ischemia, oxidative stress) to downstream damage (e.g., brain edema). The integrity of BBB directly determines the extent of fluid and toxic substance infiltration into brain tissue, thereby governing the development of cerebral edema. Thus, the following section focuses on the association between BBB integrity and brain edema.

### BBB integrity and brain edema

BBB disruption after SAH could induce varies pathophysiological processes, and the endothelial cell apoptosis was involved (23). The presence of blood breakdown products, such as oxyhemoglobin, and the associated oxidative stress have been linked to the advancement of blood–brain barrier (BBB) disruption. Additionally, the activation of inflammatory cytokines, including TNF-α, IL-1β, and thromboxane A2, can induce BBB disruption by promoting endothelial cell death and activating matrix metalloproteinases. Furthermore, various mechanisms are involved in BBB disruption, including matrix metalloproteinase-9 (MMP-9)—which degrades the extracellular matrix of the basement membrane (e.g., collagen IV, laminin) and directly cleaves tight junction proteins (occludin, ZO-1) to increase BBB permeability (23), and tight junction (TJ) proteins between endothelial cells (24). Cerebral edema may occur when blood components and inflammatory factors infiltrate the brain following disruption of the BBB. Study has indicated that aquaporin 4 (AQP4) is implicated in the development of brain edema after SAH. Inhibiting AQP4 has been associated with amelioration of cerebral edema, as it plays a crucial role in regulating water balance within brain tissue following SAH (25). To conclude, BBB disruption is the core pathogenesis of cerebral edema in EBI, with oxidative stress, inflammatory cytokines, and MMP-9 activation as key mediators. Targeting these pathways to preserve BBB integrity may effectively alleviate brain edema.

### Mechanical impact

During the acute phase of SAH, a decrease in CPP and an increase in ICP play a significant role in reducing CBF. The reasons for a decrease in CPP and increase in ICP included cerebral edema, cerebrovascular dysfunction causing congestion, and hematomas blocking cerebrospinal fluid circulation, and the levels could return normal or slightly elevated quickly. However, the pathophysiological mechanisms, such as disruption of the BBB, constriction of microvessels, formation of microthrombi, and impaired autoregulation, prolonged the time needed for the recovery of cerebral blood flow (12). Study found the arterioles’ reactivity to endothelin-1 was increased after blood injection (26). Furthermore, ultrastructural examination revealed the presence of partially collapsed capillaries, enlarged astrocyte foot processes, and protrusions from endothelial cell lumens within 1 h following SAH (27).

The progression of microthrombi can be influenced by platelets, with arterial damage and active bleeding playing a significant role in promoting platelet aggregation and the formation of microthrombi. The potential mechanism involved the liberation of serotonin, ADP, and platelet-derived growth factor subsequent to SAH, leading to potential alterations in cerebral perfusion. Moreover, the collagenases release, such as MMP-9 was associated with BBB disruption, while the inflammatory damage could exacerbate via lymphatic cells adhering to microvessels. Furthermore, the brain energy metabolism included oxygen and glucose metabolism. The decrease in cerebral metabolic rate of oxygen (CMRO_2_) after SAH is significantly related to lower CBF and elevated ICP (28). An increase in high-glucose anaerobic glycolysis has been found to be associated with a decline in neurological function score, particularly in relation to anaerobic metabolism (29).

### Ions disturbance

After SAH, there are several alterations observed in the brain microenvironment, including fluctuations in intracellular levels of calcium, potassium, sodium, and magnesium. These dynamic changes are closely linked to brain tissue injury, such as disruption of nerve electrical activity, constriction of blood vessels, and the occurrence of chronic delayed effects. Nowadays, the etiology of cerebral ischemic injury after SCH was considered induce cortical spreading depression (CSD) caused by ion imbalance combined with the secondary cortical spreading cerebral ischemia (CSCI). Possible targets for intervention to address ion imbalance involve increasing the threshold for CSD by enhancing NO availability, utilizing magnesium ion antagonism, NMDA receptor antagonists, or maintaining high perfusion pressure, all of which have been associated with shorter duration of CSD (30–33).

### Molecular alterations

The immune-inflammatory response plays a significant role in brain injury and CVS following SAH. Additionally, the activation of inflammatory cells, increased expression of immune molecules, and release of inflammatory mediators have been observed following disruption of the BBB, which may be a response to the presence of blood-derived antigens—referring to blood components (e.g., hemoglobin breakdown products, platelet-derived factors) that are normally sequestered by the BBB and enter brain tissue after disruption. It should be clarified that this inflammatory response is not a traditional antibody–antigen specific immune reaction, but rather a result of blood components acting as damage-associated molecular patterns (DAMPs) to activate innate immune pathways (34). Studies has indicated that during the acute stage of SAH, there is an increase in the levels of IL-1, IL-6, and TNF-α, and the production of these inflammatory factors involves the activation of the Ras-MAPK-NF-κB, JAK/STAT, and TLR4/NF-κB pathways (35–37).

Oxidative stress can be triggered by the breakdown products of hemoglobin that are released into the subarachnoid space following a SAH. This oxidative stress can be further intensified by cerebral ischemia–reperfusion. Oxidative stress leads to lipid peroxidation and protein oxidation, which are associated with DNA damage, cell apoptosis, and the activation of inflammatory cascade reactions. Oxidative stress induces endothelial cell apoptosis, degrades tight junction proteins, and damages the basement membrane, directly exacerbating BBB disruption (38). Thus, interventions targeting oxidative stress (e.g., inhibiting ROS production, activating antioxidant pathways) can protect endothelial function, maintain TJ integrity, thereby alleviating BBB disruption and ultimately reducing EBI severity (39).

The production of reactive oxygen species (ROS) following SAH is mediated by heme oxygenase, nicotinamide adenine dinucleotide phosphate (NADPH) oxidase, and nitric oxide synthase (NOS). NO generated by NOS was significantly reduced owing to acute cerebral vasospasm (CVS)—a pathological vasoconstriction of cerebral arteries after SAH, whereas it was dramatically increased after 24 h. Thus, NOS and NO contributed a crucial role in brain damage caused by oxidative stress (40). Studies have shown that reducing the factors that lead to the production of ROS and inhibiting ROS generation can result in enhanced antioxidant responses, which are correlated with reduced oxidative stress-induced damage (41, 42). Endothelial nitric oxide synthase (eNOS) knockout is associated with larger infarct size in cerebral infarction models (43), but in SAH, eNOS knockout is associated with less microvascular damage and exerts neuroprotective effects (44).

## BBB disruption after SAH

Disruption of the BBB, a prominent pathological feature, is strongly correlated with unfavorable outcomes following SAH. Unlike ischemic stroke (mainly caused by focal ischemia) and intracerebral hemorrhage (mainly caused by extravasated blood components), BBB disruption after SAH is multifactorial: it results from transient global cerebral ischemia after aneurysm rupture, delayed cerebral ischemia, and the toxic effects of extravasated blood components (45–47).

BBB disruption leads to the infiltration of blood components and inflammatory factors into brain tissue, triggering cerebral edema and neuroinflammation. Approximately 8–67% of SAH patients are diagnosed with global cerebral edema on admission, and nearly 12% develop delayed cerebral edema within 2 weeks (48). Elevated BBB permeability and global cerebral edema in the acute phase of SAH are significantly associated with poor prognosis and are considered important manifestations of EBI (48). Additionally, SAH comorbidities (e.g., infection, electrolyte disturbance) can further worsen BBB disruption and clinical outcomes (45).

## BBB component dysfunction and its association with EBI

**Figure 2:**
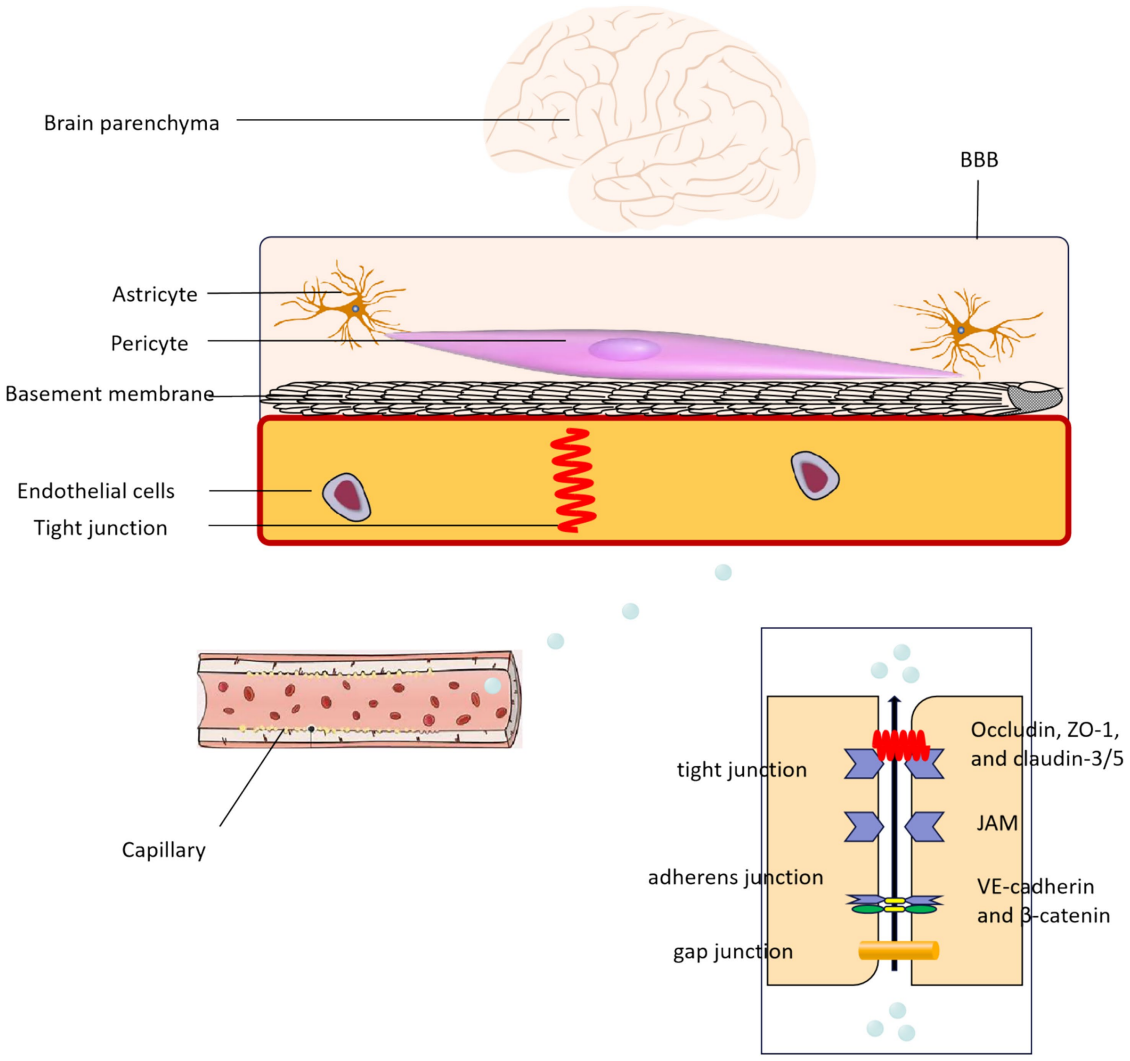
The structure and related components of the BBB.

The BBB serves as the interface between the bloodstream and the central nervous system, facilitating the communication of substances from the peripheral blood to the brain (49). The key constituents of the BBB comprise microvascular endothelial cells, TJs, astrocytes, and pericytes. The integrity and functionality of the BBB are influenced by these components (Figure 2) (50). Moreover, the components of the BBB can undergo modifications based on the specific needs of various brain regions and blood vessels. These modifications encompass alterations in glial cells, the extracellular matrix, and VSMC (51).

In addition to the classic components of the blood–brain barrier (BBB), emerging evidence in recent years has highlighted the critical role of the glymphatic drainage system in brain waste clearance (52). This system facilitates the elimination of neurotoxic substances β-amyloid (Aβ) through glymphatic fluid flow mediated by aquaporin-4 (AQP4) expressed in astrocytic end-feet (53). Following subarachnoid hemorrhage (SAH), disruption of AQP4 polarity may impair glymphatic function, leading to Aβ accumulation (54). Similar mechanisms have been observed in Alzheimer’s disease (AD), where glymphatic dysfunction is recognized as an early pathological hallmark (55). Thus, SAH-induced early brain injury may accelerate neurodegenerative progression by interfering with glymphatic clearance pathways (56).

### Microvascular endothelial cell dysfunction and EBI

Microvascular endothelial cells, which are connected by TJs, adherens junctions, and gap junction proteins, constitute the primary constituent of the BBB and serve as the initial protective barrier in the brain (57–60). Cerebral endothelial cells establish a barrier through the expression of TJ proteins, which limit vesicle-mediated transcellular transport or transporter-mediated transport (61). These cells facilitate the transportation of proteins and molecules to reach brain tissues, and the activity of vesicle transport in endothelial cells as well as the components of the plasma membrane can influence transcellular transport (62–64). Endothelial cells are widely used in BBB models, but the transporters and cellular machinery differ between polarized endothelial cells’ luminal and abluminal sides (62).

The BBB disruption caused by SAH was associated with the progression of EBI and cerebral vasospasm (CVS), depending on the response of endothelial cells (65, 66). Within 24 h following SAH, there is evidence of the presence of oxyhemoglobin, excessive iron, and oxidative stress resulting from the breakdown of red blood cells. These factors are known to contribute significantly to the apoptosis of endothelial cells (67–69). The free radicals induced by oxidative stress could cause cellular damage by accelerating DNA fragmentation, protein breakdown, and lipid peroxidation. These changes are significantly related to pathological changes in endothelial cells, leading to increased permeability of the BBB (70–73). Disruption of the basal lamina and development of cerebral vasospasm (CVS) contribute significantly to the advancement of EBI through endothelial cell damage (74, 75). Nevertheless, there are mechanisms that can suppress endothelial cell death. The presence of ApoE has a strong correlation with the inhibition of EBI in SAH. ApoE levels increase significantly after 6 h, reach their peak after 48 h, and return to normal after 72 h in SAH. This elevation of ApoE may hinder the activation of the inflammatory cyclophilin A (CypA)-NF-κB-MMP-9 pathway, thereby preserving the integrity of the BBB (76). Furthermore, the Janus kinase 2 (JAK2)/STAT3 signaling pathway has been found to partially regulate endothelial cell apoptosis, and activation of the JAK2/STAT3 cascade can result in increased expression of anti-apoptotic genes such as B-cell lymphoma 2 (Bcl-2) and Bcl-xL (77–79).

The activation of the Nrf2-ARE signaling pathway in endothelial cells following SAH is crucial for preserving BBB integrity by inhibiting endothelial cell apoptosis and countering the effects of oxidative stress. This pathway regulates the expression of detoxifying enzymes and antioxidative proteins, thereby mitigating the detrimental effects of SAH-induced oxidative stress on the BBB (80–82). The level of v-erb-b2 avian erythroblastic leukemia viral oncogene homolog 4 (ErbB4) is upregulated in endothelial cells 72 h after SAH. ErbB4 triggers the activation of the yes-associated protein (YAP)/phosphatidylinositol-4,5-Bisphosphate 3-Kinase Catalytic Subunit beta (PIK3CB) signaling pathway, resulting in enhanced expression of occludin and claudin-5. This pathway promotes endothelial cell survival under oxidative stress and stabilizes intercellular junctions, thereby maintaining BBB integrity and reducing brain edema (83).

The integrity of BBB could be affected by the endothelial cytoskeleton. Studies has demonstrated that increased expression of myosin light chain kinase (MLCK) is linked to heightened phosphorylation of myosin light chain (MLC), resulting in reorganization of the cytoskeleton, disrupted cell–cell interactions in endothelial cells, compromised BBB integrity, and the development of vasogenic brain edema following SAH (84). Moreover, the integrity of endothelial TJs plays a crucial role in preventing platelet adhesion to extracellular collagen, thus maintaining the delicate equilibrium between hemostasis and thrombosis. However, the dysregulation of this equilibrium occurs due to the upregulation of vascular endothelial growth factor (VEGF) expression following vasospasm subsequent to SAH (85–87). In the acute phase of SAH, the increased expression of VEGF may impact platelet adhesion and disruption of endothelial TJ through the regulation of collagen IV exposure and its binding to platelet glycoprotein Ia-II (88, 89). These alterations can affect the entry of platelets into the brain, triggering neuroinflammation and EBI following SAH (90–92). Furthermore, insufficient NO production by endothelial cells failed to effectively inhibit platelet adhesion and aggregation, thus potentially contributing to the development of ischemic brain injury following SAH (93). The enhanced protein kinase C (PKC) expression was regarded as the primary mechanism for vasospasms, and the PKC family was significantly related to S100 calcium-binding protein during cerebral vasospasm (CVS) after SAH (94). SAH could change transport mechanisms among endothelial cells, and P-glycoprotein (P-gP) was reduced after SAH (95). Vesicular trafficking in endothelial cells and changes in Mfsd2a expression and TJ proteins have a significant impact on BBB permeability following SAH (96).

Studies have already demonstrated that BBB integrity could be assessed using the von Willebrand factor (vWF), thrombomodulin (TM), and endothelin 1 (ET-1). The expression of these markers increases SAH, demonstrating disrupted BBB (97). The p38 MAPK-p53/NF-κB (p65) signaling pathway involved TM’s role on endothelial TJ proteins after SAH (98). Moreover, the expression of ET-1 could affect the role of endothelial cells on VSMC (99). In vascular smooth muscle cells, the binding of ET-1 to the ETA receptor can trigger the activation of the ERK1/2 pathway and the Kruppel-like transcription factor 4 (KLF4), leading to the transition of VSMCs from a contractile phenotype to a synthetic phenotype, thereby inducing KLF4 activation (100, 101). Studies have indicated that the expression level of endothelin-1 increases after subarachnoid hemorrhage, and the activation of endothelial nitric oxide synthase (eNOS) can provide negative feedback on the expression of endothelin-1 (102). In addition, lipoxin A4 (LXA4) plays a vital role in endothelial cells by inhibiting neutrophil infiltration and pro-inflammatory cytokines. It exerts regulatory control over the NF-κB signaling pathway through the inhibition of ERK1/2 phosphorylation, resulting in reduced levels of pro-inflammatory cytokines and decreased neutrophil infiltration (103).

### Tight junction disruption and EBI

The TJs, adherens junctions, and gap junctions constituted the intercellular junctional complexes among endothelial cells (64). TJs are composed of several proteins, such as occludin, zonula occludens-1 (ZO-1), and claudin-5 (104). These TJ proteins exhibited structural similarity as phosphoproteins, and their interaction, redistribution, and transmembrane protein localization could be modified through phosphorylation (104, 105). The TJ system, which is a part of the immunoglobulin superfamily, includes the junctional adhesion molecules (JAMs). JAMs play a role in facilitating leukocyte migration across endothelial cell layers through homotypic and heterotypic interactions with other members of the JAM family. These interactions have an impact on endothelial cells (106). Furthermore, the disassembly of TJs was associated with an elevated BBB permeability, then the BBB disruption was occurred, which could causing brain edema formation after SAH. The transport of materials across the BBB includes paracellular and transcellular pathways. The paracellular pathway depends on the integrity of intercellular junctions (e.g., TJs, adherens junctions)—SAH-induced TJ degradation opens this pathway, allowing macromolecule leakage. The transcellular pathway is mediated by vesicular transport (e.g., receptor-mediated endocytosis)—SAH activates abnormal vesicular trafficking, further increasing BBB permeability (64).

The expression of TJ proteins (claudin-3 and claudin-5) can be regulated by sphingosine-1-phosphate receptor-1 (S1P1) proteins, which activate the PI3K/Akt signaling pathway. This activation leads to the inhibition of glycogen synthase kinase 3 β (GSK3β) and the stabilization of β-catenin. S1PI is primarily located in endothelial cells, and its expression level decreases 24 h after SAH, leading to changes in TJ protein expression (107). The activation of S1P1 by PAR-1 involves the regulation of endothelial protein C receptor (EPCR) and activated protein C (APC) (98). The expression of adhesion molecules on the luminal surface of endothelial cells was increased in response to the presence of blood in the subarachnoid space (108–112). These molecules facilitate the communication between endothelial cells and leukocytes, promoting the recruitment, adhesion, and migration of white blood cells to the site of bleeding (113–115). In addition, it has been observed that neutrophils infiltrate the brain within the first 10 min following SAH, which is strongly associated with a decrease in cerebral NO levels due to the activity of the neutrophil-derived enzyme myeloperoxidase. Research studies have indicated that neutrophil infiltration contributes to the disruption of the BBB after SAH by releasing ROS, elastases, proteases, collagenase, and matrix metalloproteinase-9 (MMP-9) (116).

The disruption of TJs between endothelial cells triggers the activation of the NF-κB inflammatory signaling pathway, resulting in the development of posthemorrhagic vasogenic edema (117). The primary cause of EBI observed within 24 h after SAH is believed to be alterations in the expression levels of TJ proteins. Moreover, a decrease in the levels of various TJ proteins, such as ZO-1, occludin, claudin-5, JAM-A, and adherens junction protein VE-cadherin, has been observed between 24 and 48 h following SAH (118). Studies have reported a significant correlation between elevated permeability and reduced expression levels of tight at 3 h and 72 h after SAH (104). T2-weighted MRI hyperintensities could be observed after SAH, indicating increased BBB permeability in the early stages of SAH (89, 119). The loss of collagen IV mediates perturbations in the microvascular basal lamina after SAH, promoting the progression of BBB disruption. The levels of MMP-9 and collagenase activity reach their highest point 3–6 h after SAH, whereas the expression of collagen IV decreases in two distinct phases (13, 120). The alterations in collagen IV expression paralleled the biphasic alterations observed in the TJ proteins ZO-1 and occludin (104). Collagen IV decreases in two phases: first at 3–6 h post-SAH (driven by initial MMP-9 activation) and second at 24–48 h (due to sustained inflammation), consistent with TJ protein changes. Furthermore, laminin—a substrate for MMP-9—decreases at 24 h post-SAH, and endothelial MMP-9 upregulation coincides with reductions in laminin, occludin, and collagen IV (121–123).

The NF-κB-MMP-9 molecular signaling pathway is implicated in the regulation of pathophysiological cascades within cerebral endothelial cells following SAH (124). Inflammation contributes to the induction of BBB disruption, as indicated by the increased expressions of toll-like receptor (TLR)-4 and high-mobility group box 1 (HMGB1) following SAH (125, 126). Furthermore, the elevation of MMP-9 levels can be triggered by the activation of p53 expression through the NF-κB signaling pathway in brain endothelial cells 24 h post-SAH. This activation can lead to the degradation of collagen IV and laminin, subsequently resulting in the degradation of occludin and disruption of the basal lamina (127). The inflammatory-induced disruption of TJ proteins contributes to the onset of vasogenic brain edema 24 h following SAH (128).

### Astrocyte activation and EBI

Astrocytes, which are the predominant cell type in the central nervous system, play crucial roles in the maturation, viability, metabolic support, and neurotransmission of neuronal activities (129). They maintain and repair the BBB by releasing various effector molecules that induce barrier properties, transporter polarization, and overall BBB function (130–132). Astrocytes also serve as a cellular link between neuronal activity and blood vessels, modulating cerebral blood flow and brain water content in response to neuronal activity (133, 134). Astrocytic end-feet undergo polarization—functional proteins (e.g., AQP4) are directionally distributed on the membrane contacting vessel walls. This polarization is regulated by pericyte-derived signals, forming a functional interface of the BBB (135).

After SAH, astrocytes exhibit morphological alterations characterized by dilated end-feet and endothelial protrusions—these changes compress capillary lumens and disrupt cerebral ultrastructure (136). These changes lead to compression of the capillary lumen and disruption of cerebral ultrastructure (136). Astrocytes react to damage-associated molecular patterns (DAMPs) originating from the perivascular area, resulting in the production of pro-inflammatory cytokines, chemokines, growth factors, as well as the attraction and activation of immune cells from the periphery (137). Astrocytes express Toll-like receptor 4 (TLR4), which plays a crucial role in the progression of neuroinflammation (138–140). This function is exemplified by the increased expression of myeloid differentiation primary response protein 88 (MyD88), a crucial mediator for delivering TLR signals to NF-κB (141). Stimulation of the TLR4/MyD88 signaling pathway leads to the ubiquitination of tumor necrosis factor receptor-associated factor 6 (TRAF6). The ubiquitylation of TRAF6 leads to the degradation of ULK1 or the prevention of ULK1 phosphorylation. This process exacerbates brain injury after SAH by inhibiting autophagy (142, 143). Activation of NF-κB can trigger the upregulation of p65, TNF-α, and IL-1β via the TLR4/MyD88 signaling pathway. The PI3K/Akt signaling pathway, which is downstream of the angiogenic factor with G-patch and FHA domain 1 (Aggf1), attenuates the expression of these pro-inflammatory molecules. Aggf1 in astrocytes exerts a significant influence on the PI3K/Akt signaling pathway following SAH (144).

Astrocytes have the ability to differentiate into two distinct phenotypes following SAH: the pro-inflammatory/harmful A1 phenotype and the anti-inflammatory/ beneficial A2 phenotype (145). The A1 polarization in response to SAH is triggered by the activation of microglia and the release of pro-inflammatory cytokines (146, 147). TNF-α induces astrocyte differentiation towards the detrimental A1 phenotype via NF-κB activation. Conversely, TNF-α promotes astrocyte differentiation towards the advantageous A2 phenotype through the induction of neuronal-derived prokineticin 2 (PK2) expression, activating the STATA3 cascade (148). Moreover, the diminished capacity of astrocytes to remove glutamate from the synaptic cleft following SAH is attributed to the decreased expression of glutamate transporter 1 (GLT-1) and EAAT-2 on the astrocytic membrane, leading to subsequent neuronal harm (149). The SAH-associated decrease in Akt phosphorylation could explain this decrease in EAAT-2 expression of GLT-1, which could be affected by histone deacetylase 2 (HDAC2) (150). These alterations may exert a detrimental effect on hippocampal synaptogenesis and lead to cognitive impairment following SAH (151).

Astrocytes play a role in brain damage via an elevated ET-1 expression after SAH, as they are the major source of ET-1 production (152, 153). However, ET-1 exerts positive effects by promoting the synthesis of BDNF, GDNF, and NT3 (100)—these neurotrophic factors support neuronal survival, axon regeneration, and synaptic remodeling, offsetting SAH-induced neuronal damage. Furthermore, the levels of glial fibrillary acidic protein (GFAP) and heme oxygenase 1 (HO-1) were found to be elevated in astrocytes following SAH. This increase can be attributed to the passage of PDGF released by platelets through the endothelium and basal lamina into the brain tissue (149). Ferritin expression in astrocytes plays a cytoprotective role by attenuating neuronal Hb toxicity (154). CD163 receptors present on microglia and neurons are capable of uptaking haptoglobin-Hb complexes, thereby extracting iron from astrocytes following SAH, leading to the initiation of brain damage (154–156).

### Pericyte dysfunction and EBI

Pericytes are located on the outer surface of microvessels and are integrated into the vascular basement membrane. They establish connections with endothelial cells through various types of junctions, including gaps, adherens junctions, and peg-and-socket junctions (157, 158). The pericytes in cerebral tissue were higher than in peripheral tissues, which could regulate angiogenesis and the extracellular matrix deposition to maintain the endothelial cell monolayer (157). Moreover, pericytes were essential for tight junctions and BBB function in the brain, and pericytes’ contractility could regulate cerebral blood flow by altering capillary diameter (158–160).

Given the multifaceted functions of pericytes, which play a crucial role in the intricate pathophysiology following SAH due to their unique characteristics (161), picytes can express alpha-smooth muscle actin (α-SMA) to enhance the constriction of capillary lumens after SAH (162, 163). Additionally, pericytes with an α-SMA phenotype can release factors that compromise the integrity of the BBB (164, 165). The release of Hb from ruptured red blood cells within pericytes can trigger microvascular constriction through the scavenging of NO during the initial phase following SAH. Reduced levels of NO after SAH are strongly associated with pericyte contraction. However, during the later stages after SAH, pericyte contraction is induced by decreased expression of eNOS (166, 167).

The upregulation of MMP-9 in pericytes following SAH may be attributed to the activation of protease-activated receptors (PARs) by thrombin. This activation triggers the activation of G-protein coupled receptors and subsequent signaling through the PKCθ-Akt and PKCδ-ERK1/2 pathways (168–171). Additionally, MMP-9 expression could be induced by ROS through the activation of the NF-κB inflammatory pathway (172). Pericytes play a significant role in the pathophysiological cascade by secreting cyclophilin A (CypA), and the elevated expression of CypA was co-localized with pericyte markers (173). Moreover, greater ferritin was localized in pericytes after SAH, suggesting that pericytes store iron and are associated with low oxygen tension, high ROS, and acidosis. Fe^2+^ could accelerate ROS production under SAH or ischemic conditions and induce electrolyte imbalance (174). The DAMPs in the perivascular spaces could activate pericytes and initiate a local pro-inflammatory response, which could be related to the infiltration of leukocytes and the degradation of tight junctions (161).

### Therapeutic targets in the BBB

In preclinical models, multiple therapeutic strategies have been identified to enhance BBB integrity following SAH. However, the mechanisms underlying these treatments and their specific targets for BBB disruption have not been examined. Several molecular mediators, including MMP-9 (175), Nrf2 (176, 177), TLR4 (178, 179), VEGF (180, 181), and ZO-1 (5), have been observed to contribute to BBB disruption in SAH-induced EBI. Many studies have explored the mechanisms and possible therapeutic targets for BBB disruption in EBI following SAH, and these findings have been summarized in Table 2.

**Table 2:** Potential therapeutic targets and agents for early brain injury in the blood–brain barrier.

Components of the BBB	Molecular mediators	Potential therapeutic agents
Microvascular endothelial cells dysfunction	MMP-9	ApoE-mimetic peptide (193)
	ALOX15	Cepharanthine (194)
	Caspases	XIAP, VX-765, and Z-VAD-FMK (195)
Disruption of tight junctions	Ang-1, MAPK, VEGF-A	Recombinant osteopontin (198)
	ATF6/CHOP	Apelin-13 (209)
	RIP3/MLKL	Necrostatin-1 (211)
	VASP, occludin, AQP	JNJ16259685 (213)
	MMP-9, RIP3, MLKL	Celastrol (175)
	HIF-1α, MMP-9, and VEGF	2-Methoxyestradiol (215)
	ZO-1, caspase-3, Bax, Bcl-2, TLR2, and TLR4	Cerebrolysin (216)
Astrocytes and pericytes	APQ4	Hydrogen sulfide, β-hydroxybutyrate (54, 217)
	Nrf2, ho-1, phospho-Akt, Bcl-2, Bcl-2-related X protein	Gastrodin (219)
	STAT3	Ponesimod (221)
	Cdk5	Roscovitine (222)
	PI3K/AKT	Recombinant GBP2 protein and LY294002 (223)
	ERRγ/PGC-1α/SIRT3	DY131 (225)

### Microvascular endothelial cells dysfunction

Cerebral capillary endothelial cells maintain BBB function. However, BBB disruption could occur because of injuries to endothelial cells caused by aneurysmal rupture. Endothelial cell apoptosis may be triggered within 24 h of SAH by factors such as oxidative stress, oxyhemoglobin, and iron overload (68, 91). Oxyhemoglobin exerts cytotoxic effects on endothelial cells by activating caspase-3, caspase-8, caspase-9, and MMP-9, elevating intracellular Ca^2+^ and free radicals (68). Oxidative stress injury, excessive free radicals, extracellular hemoglobin, and iron overload following SAH were associated with several endothelial cell damage. Furthermore, the overproduction of ROS could activate proapoptotic signals, accelerating cell apoptosis and aggravating BBB disruption through p53, caspase-3, and caspase-9 (182). Furthermore, the factors implicated in endothelial cell damage following SAH encompass heme, thrombin, platelets, fibrinogen, and leukocytes, which have the potential to activate microglia and TLR4. TLR4 could recognize damage and induces inflammatory cascades through activation of NF-κB, activator protein-1, or both mediated by MAPKs (46, 141). ErbB4, a member of the EGFR tyrosine kinase family, also plays a role in regulating the guidance of axons, the outgrowth of neurites, and the signaling between synapses (183). Study has demonstrated that ErbB4 can enhance the survival of endothelial cells and maintain the integrity of the BBB in the presence of oxidative stress injury (184). The activation of ErbB4 enhances the expression of tight junction proteins, including occludin and claudin-5, and promotes protective effects through the ErbB4/YAP/PIK3CB signaling pathway (83). Focal adhesion kinase (FAK) plays a crucial role in regulating the function of endothelial cells and maintaining the integrity of the endothelial barrier. When FAK is absent in endothelial cells, it can lead to the disruption of barrier integrity and abnormal distribution of vascular endothelial cadherin (185).

EBI can occur due to the leakage of blood into the subarachnoid space and subsequent dissemination through the cerebrospinal fluid surrounding the brain. This can lead to a sudden increase in intracranial pressure, reduced cerebral blood flow, cerebral edema, acute vasospasm, global cerebral ischemia, and impaired autoregulation (186, 187). These instabilities are significantly associated with the prognosis of SAH (5, 188). The large and small cerebral vessel constriction occurs immediately after SAH, and the vasoconstriction-mediating endothelial cell receptors, endothelin B or serotonin receptors (5-HT1B), were upregulated. However, the expression of the vasodilator NO in the cerebral arteries was reduced (189–191). The hyper-responsive endothelial cells could activate smooth muscle cells (192). Several studies have illustrated the underlying molecular changes of endothelial cell dysfunction for EBI (193–195). Pang et al. (193) discovered that the administration of ApoE-mimetic peptides could effectively suppress endothelial cell apoptosis and enhance the prognosis of EBI. This treatment approach resulted in several beneficial outcomes, including decreased brain edema and neuronal apoptosis, enhanced cerebral glucose uptake, and improved neurological functions. These effects were achieved by inhibiting the pro-inflammatory activators of MMP-9. Gao et al. (194) provided evidence of ferroptosis induction in microglia and endothelial cells following SAH, accompanied by an upregulation of 15-lipoxygenase-1 (ALOX15) expression. Cepharanthine exerts an inhibitory effect on ferroptosis by suppressing the expression of ALOX15. Finally, caspases were involved in endothelial cell apoptosis at the early stages after SAH, and caspase inhibitors (XIAP, VX-765, and Z-VAD-FMK) could ameliorate EBI after SAH (195).

### Disruption of tight junctions

The integrity and permeability of the BBB may be compromised due to endothelial cell contraction and disassembly of TJs, leading to the development of brain edema following SAH (68). Substances transport across the BBB through the paracellular and transcellular pathways. The disruption of TJs may result in the leakage of exudate into the brain’s extracellular space, leading to the accumulation of extracellular fluid (196). The phosphorylation of TJ proteins can modulate the interaction, redistribution, and subcellular localization of transmembrane proteins, such as occludin, ZO-1, and claudin-5 (104). The downregulation of occludin and ZO-1 in tight junctions could cause capillary leakage, thereby increasing BBB permeability (186, 197). The role of MMP-9 in the early stages of BBB disruption after SAH has been illustrated. Inflammatory cytokines and ROS can stimulate the production of MMP-9, which can result in the breakdown of the extracellular matrix surrounding cerebral microvessels and the disruption of TJ proteins between endothelial cells (121). NF-κB activation facilitates the transcription of MMP-9 and tissue inhibitor of MMP-1. The dynamic interplay between MMP-9 and tissue inhibitor of MMP-1 plays a crucial role in determining the extent of BBB disruption following SAH (198, 199). Additionally, the activation of MAPK signaling pathway may facilitate the activation of MMP-9, while the disruption of ZO-1 in endothelial cells could lead to the upregulation of tenascin-C and periostin matricellular proteins (200–202). Furthermore, galectin-3 could activate MMP-9 in endothelial cells and affect BBB disruption through the MAPK signaling pathway (203, 204).

Heat shock protein 70 (HSP 70) significantly reduces MMP-9 activity, and BBB disruption and cell death could be mediated via aberrant proteolysis, which regulates inflammation and brain edema during EBI (205). Moreover, NF-κB and MAPKs could be activated by TLR4 after SAH, resulting in the upregulation of pro-inflammatory cytokines and mediators (201). Studies have demonstrated that several molecules can be stimulated at different phases of BBB disruption, such as VEGF-A, VEGF-B, angiopoietin-1 (Ang-1), angiopoietin-2 (Ang-2), MAPKs, and MAPK phosphatase-1 (206). VEGF-A has the potential to disrupt the BBB, whereas VEGF-B and Ang-1 have the ability to counteract the effects of VEGF-A by regulating MAPK signaling pathway. MAPK phosphatase-1 is recognized as an intrinsic inhibitor of MAPK, while Ang-2 functions as an endogenous antagonist of Ang-1 (207, 208). Thus, the decrease in Ang-1 and MAPK phosphatase-1 expression, along with the increase in MAPKs and VEGF-A expression, contributed to the disruption of the BBB following SAH. Conversely, the upregulation of MAPK phosphatase-1, inactivation of MAPKs, and downregulation of VEGF-A could ameliorate BBB damage (206). These molecules could be regulated by recombinant osteopontin to restore BBB function, whereas Ang-2 and VEGF-B were not altered (206). Additionally, BBB disruption could be regulated by the hypoxia-HIF-1α-LCN2-VEGF-A signaling pathway (119, 208). The expression of activating transcription factor 6 (ATF6) is strongly associated with BBB disruption and is increased during EBI. On the other hand, the administration of apelin-13 has been shown to suppress the ATF6/CHOP signaling pathway, thereby ameliorating BBB damage (209). The activation of the Wnt/β-catenin signaling pathway could be a potential therapeutic target for enhancing the integrity of the BBB. β-catenin, an adherens junction protein and a transducer of the Wnt pathway, plays a role in the endogenous protective mechanism that regulates BBB function. Disruption of β-catenin is associated with decreased levels of tight junction proteins, which are crucial for maintaining BBB integrity. However, activation of the Wnt/β-catenin signaling pathway can attenuate this disruption and promote the expression of tight junction proteins, thereby improving BBB integrity (210). The tropomyosin-related kinase receptor B (TrkB) activation could ameliorate EBI by restoring the TJ protein ZO-1 via the activated Wnt/β-catenin signaling pathway (196).

Recent studies have elucidated the underlying molecular changes in tight junction disruption during EBI. The utilization of necrostatin-1, a selective inhibitor of receptor-interacting protein kinase 1 (RIP1), may mitigate albumin leakage and degradation of TJ proteins, serving as a neuroprotective agent following SAH through the inhibition of RIP3/MLKL signaling pathway activity (211, 212). The administration of the metabotropic glutamate receptor 1 (mGluR1) negative allosteric modulator JNJ16259685 via intraperitoneal injection at 72 h post-SAH could lead to an increase in phosphorylation of the vasodilator-stimulated phosphoprotein (VASP) and occludin, as well as a reduction in aquaporin (AQP) levels (213). In addition, celastrol has the potential to improve the leakage of albumin and degradation of TJ proteins in order to preserve the integrity of the BBB. This is achieved by inhibiting the expression of MMP-9 and exerting anti-neuroinflammatory effects. Furthermore, celastrol has the ability to decrease the levels of RIP3 and MLKL, which are proteins associated with necroptosis, and reduce the presence of propidium iodide-positive cells in the basal cortex (175). The inhibition of NLRP3 was linked to reduced cerebral edema, preservation of tight junction integrity, prevention of microthrombosis, and modulation of microglial reactive morphology (214). Hu et al. (215) discovered that the administration of 2-Methoxyestradiol demonstrated a potential to ameliorate the disruption of tight junction proteins and suppress the expression of HIF-1α, MMP-9, and VEGF. Consequently, this led to an improvement in the inflammatory response to EBI and BBB disruption following SAH. Finally, cerebrolysin could upregulate ZO-1 levels, reduce the protein expression of caspase-3 or Bax, and increase the Bcl-2 expression level. The TLR2 and TLR4 levels were also reduced after cerebrolysin treatment (216).

### Astrocytes and pericytes

Astrocytes and pericytes are crucial for maintaining the integrity of the BBB. However, there is limited research on the mechanisms and potential therapeutic targets involving astrocytes and pericytes in EBI following SAH. Hydrogen sulfide has the potential to reduce the formation of brain edema by protecting the BBB and reducing the expression of AQP4 on astrocytes through the inhibition of glial cell activation and the secretion of pro-inflammatory cytokines (217).

Moreover, the pathological process of EBI after SAH could be affected by Nox4, whereas there is no overlay effect of Nox2 inhibition and Nox4 inhibition for preventing EBI after SAH (218). SAH could induce microglial activation, astrocyte activation, and neuronal apoptosis, and the use of gastrodin could suppress these molecular changes. Additionally, it could reduce oxidative stress and inflammatory response, upregulate the Nrf2, ho-1, phospho-Akt, or Bcl-2, and downregulate the Bcl-2-related X protein and cleaved caspase-3 (219). Promoting PK2 expression and utilizing recombinant PK2 can selectively regulate astrocytic polarization, inducing a protective phenotype following SAH (148). The upregulated expression of the calcium-sensing receptor in neurons, astrocytes, and microglia after SAH could promote EBI via the CaMKII/NLRP3 signaling pathway (220). Furthermore, ponesimod could prevent astrocytic polarization to the A1 phenotype, and ponesimod could mediate astrocytic response via the STAT3 signaling pathway (221). The expression of cyclin-dependent kinase 5 (Cdk5) was observed in both neurons and astrocytes following SAH. Treatment with roscovitine resulted in the improvement of EBI and reduction of cerebral edema after SAH by inhibiting the activity of Cdk5 (222). The 6-gingerol could activate PI3K/AKT signaling pathway, and recombinant GBP2 protein and LY294002 (PI3K inhibitor) could reverse the effects of 6-gingerol (223). Furthermore, miR-26b may exacerbate EBI and the inflammatory response following SAH, promoting hemoglobin-induced apoptosis in astrocytes. Conversely, the suppression of miR-26b could alleviate EBI after SAH by modulating the KLF4/STAT3/HMGB1 signaling pathway (224). Activation of Estrogen-related receptor γ (ERRγ) using DY131 may enhance the reduction of oxidative stress and neuronal apoptosis following SAH through the ERRγ/PGC-1α/SIRT3 signaling pathway. This pathway could potentially be targeted as a novel therapeutic approach to mitigate EBI after SAH (225).

The regulatory pathways of pericyte involved in SAH include the PDGF pathway (226), the Notch pathway (227), the canonical Wnt/β-catenin pathway (228), and new players in the pericyte signaling pathway, such as miRNA, exosome, and lncRNA (161). Moreover, the pericyte α-SMA phenotype could inhibit the NO/cGMP signaling pathway to mediate acute microvessel constriction after SAH, and the eNOS or the pericyte α-SMA phenotype could be considered therapeutic targets (167). ApoE deficiency could activate the CypA-NF-κB-MMP-9 pathway and induce the degradation of N-cadherin, resulting in greater pericytes loss, which is significantly associated with EBI after SAH (229). Furthermore, the glial limitans formation and neurological function could be regulated by pericytes through EphA4/EphrinB2 signaling pathway, and the intervention should be applied as a novel treatment target for improving EBI after SAH (230).

### Glymphatic system impairment and EBI

Subarachnoid hemorrhage (SAH) impairs meningeal lymphatic system function. Evaluations using gadopentetate dimeglumine distribution reveal reduced cerebrospinal fluid inflow post-SAH, more prominently in the ipsilateral hemisphere, persisting for 1 week with partial recovery by the second week. Under physiological conditions, gadopentetate dimeglumine rapidly fills regions like the olfactory bulb and optic nerve, while SAH induces delayed and diminished outflow in these areas, indicating lymphatic clearance dysfunction. Additionally, intermittent cerebellomedullary cistern cerebrospinal fluid drainage markedly improves glymphatic and meningeal lymphatic system function following SAH (56).

A study integrating single-cell RNA sequencing, spatial transcriptomics, and *in vivo*/*in vitro* experiments delineated the spatial and cellular alterations of meningeal lymphatic vessels (mLVs) in the early phase after SAH. It demonstrated that THBS1 overexpression and its interaction with CD47 may induce meningeal lymphatic endothelial cell (mLEC) apoptosis via the STAT3/Bcl-2 signaling pathway. Concurrently, S100α6 was identified as associated with poor prognosis, potentially serving as a novel biomarker for meningeal lymphatic injury, with a linear relationship to THBS1 expression (231).

Th17 cells are linked to post-SAH brain injury, though their cerebral clearance mechanism remains unclear (232). SAH model mice exhibit significant behavioral abnormalities, brain injury, and cerebral immune cell accumulation. Further investigations show that laser ablation of the meningeal lymphatic system or CCR7 knockout leads to meningeal Th17 cell accumulation, reduced neurological scores, and elevated inflammatory factor levels. Conversely, VEGF-C or CCL21 protein injection promotes Th17 cell drainage to lymph nodes, improves neurological scores, and lowers inflammatory factor levels (233).

Another study reported increased aquaporin 4 (AQP4) expression post-SAH (234). Notably, SNTA1 knockout reduces AQP4 polarization, implying a association between AQP4 polarization and SNTA1 expression (235). Western blot and immunofluorescence analyses indicated that post-SAH, AQP4 expression increases while polarization decreases, with concurrent SNTA1 upregulation. BHB treatment restores AQP4 polarization by upregulating SNTA1, enhances meningeal lymphatic system function, mitigates neuroinflammation, and thereby ameliorates neurological deficits in SAH mice (54).

Meningeal lymphatic vessels also play a key role in Alzheimer’s disease (AD) by modulating β-amyloid (Aβ) clearance, microglial activation, and immunotherapeutic efficacy (236). Post-SAH early brain injury and delayed cerebral ischemia trigger neuroinflammatory responses, which are closely linked to AD neurodegeneration (237, 238). Deep cervical lymphatic-venous anastomosis (LVA) is safe and effective, significantly improving AD patients’ cognitive function as evidenced by pre- and post-surgical Mini-Mental Status Examination, though its long-term efficacy requires verification through large-scale clinical trials and prolonged follow-up (55, 239).

## Perspective

There are multiple pathological and molecular processes that contribute to the breakdown of the BBB following SAH, while the precise mechanisms and potential therapeutic targets for EBI after SAH in the BBB are still not fully understood. Currently, the majority of therapeutic interventions aim to enhance endothelial cell dysfunction and restore TJ integrity following SAH, but these interventions have not yet been successfully translated into clinical practice. Moreover, the complex pathogeneses of BBB disruption after SAH and its role in causing EBI makes it difficult to alleviate by inhibiting a single pathway or molecule. Therefore, it is essential to investigate the underlying mechanisms and identify potential therapeutic molecular targets for the BBB to enhance EBI following SAH. Additional studies should be performed to identify potential therapeutic targets against BBB disruption and improve the EBI after SAH. Notably, the neurodegenerative process in Alzheimer’s disease (AD) is closely linked to that following subarachnoid hemorrhage (SAH). Deep cervical lymphatic-venous anastomosis (LVA), as an emerging therapeutic technique for improving cognitive function in AD patients, remains to be validated through large-scale clinical trials and long-term follow-up investigations.
